# Species Delimitation in the Genus *Moschus* (Ruminantia: Moschidae) and Its High-Plateau Origin

**DOI:** 10.1371/journal.pone.0134183

**Published:** 2015-08-17

**Authors:** Tao Pan, Hui Wang, Chaochao Hu, Zhonglou Sun, Xiaoxue Zhu, Tao Meng, Xiuxiang Meng, Baowei Zhang

**Affiliations:** 1 Anhui Key Laboratory of Eco-engineering and Bio-technique, School of Life Sciences, Anhui University, Hefei, 230601, Anhui, China; 2 School of Life Science, Nanjing Normal University, Nanjing, 230039, Jiangsu, China; 3 Guangxi Forest Inventory and Planning Institute, Nanning, 530011, Guangxi, China; 4 School of Environment and Natural Resources, Renmin University of China, Beijing, 100872, China; Sichuan University, CHINA

## Abstract

The authenticity of controversial species is a significant challenge for systematic biologists. Moschidae is a small family of musk deer in the Artiodactyla, composing only one genus, *Moschus*. Historically, the number of species in the Moschidae family has been debated. Presently, most musk deer species were restricted in the Tibetan Plateau and surrounding/adjacent areas, which implied that the evolution of *Moschus* might have been punctuated by the uplift of the Tibetan Plateau. In this study, we aimed to determine the evolutionary history and delimit the species in *Moschus* by exploring the complete mitochondrial genome (mtDNA) and other mitochondrial gene. Our study demonstrated that six species, *M*. *leucogaster*, *M*. *fuscus*, *M*. *moschiferus*, *M*. *berezovskii*, *M*. *chrysogaster* and *M*. *anhuiensis*, were authentic species in the genus *Moschus*. Phylogenetic analysis and molecular dating showed that the ancestor of the present Moschidae originates from Tibetan Plateau which suggested that the evolution of *Moschus* was prompted by the most intense orogenic movement of the Tibetan Plateau during the Pliocene age, and alternating glacial-interglacial geological eras.

## Introduction

Speciation, extinction and migration are often driven by historical, ecological and biogeographic factors, which have played important roles in shaping global biodiversity by influencing regional differentiation [1,2]. In Asia, the uplift of the Tibetan Plateau was the most remarkable geological event because of the average altitude of the Tibetan Plateau, which was raised by approximately 3,000 m during the Quaternary period [3,4]. Associated with the uplift of the Tibetan Plateau, high mountains and deep valleys were generated, which profoundly accelerated ecological speciation events [5,6,7]. In the meantime, the uplift of the Tibetan Plateau led to the desertification of northern China because of the obstruction of the northward flow of warm and wet-air from India across the mainland of China [8,9,10], which resulted in a greater impact across a much larger spatial scale.

Accompanying the above process, global climatic oscillation in the late Pleistocene age (started approximately 2.8 million years ago) also made a significant difference to endemic species of the Tibetan Plateau [11]. To a large extent, cyclical climatic changes and alternating glacial-interglacial geological periods determined current spatial distribution and genetic structures of many species [12,13]. In addition, some studies showed that intraspecific divergence in many Tibetan Plateau endemic species were influenced by Pleistocene age glacial cycles [14,15,16,17]. However, more details are still needed to strengthen our understanding of the historical, ecological and biogeographical factors that influence speciation, extinction and migration events. Recently, the discovery of some megaherbivore fossils (e.g., *Coelodonta thibetana*, *Pseudois nayaur*, and *Ovis ammon*) in the Tibetan Plateau suggest that some herbivore species may be a high plateau origin species spanning 6.4–5.3 Ma [11,18]. Therefore, it is interesting to identify the relationship between speciation processes and paleo-environmental changes during this period [19,20].

**Fig 1:**
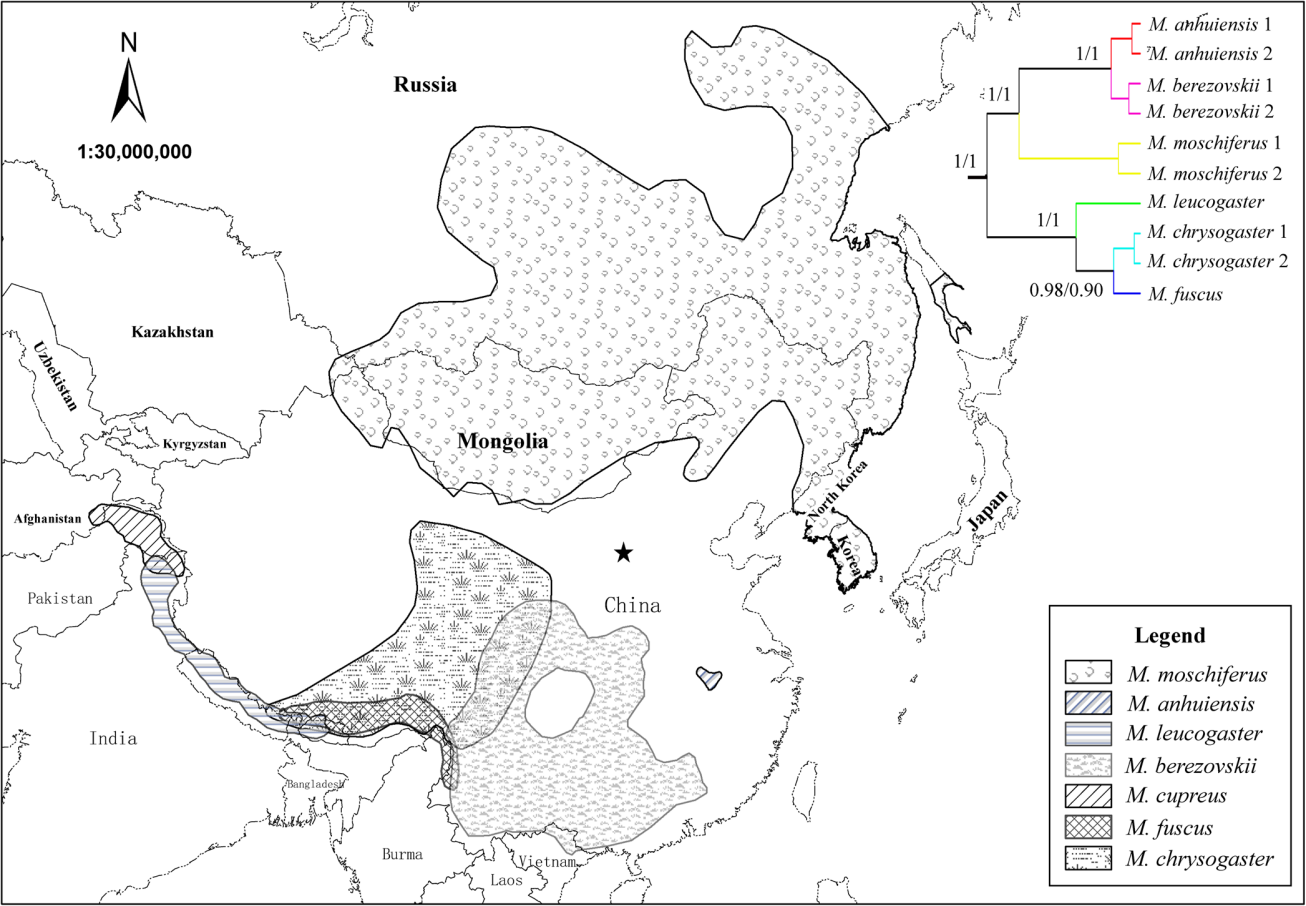
Geographic distribution of *Moschus* species and consensus mitochondrial gene tree. Tree is equivalent to that of Fig 3. All the information about geographic distribution of *Moschus* species were came from IUCN (http://www.iucnredlist.org/), except a new distribution area of *M*. *berezovskii*, which was marked by a star [39].

Musk deers (*Moschus*, Moschidae) are endemic to the Palearctic era, which are mostly distributed around the Tibetan Plateau, its adjacent mountainous area in China and Far East Area (Fig 1). The fossils of *Moschus* were found in China, Northern India, Mongolia and Uzbekistan [21]. In 1821, Moschidae was established by Gray based on specimen of *M*. *moschiferus* [22,23]. Up to now, there are seven species were that are described in Moschidae [24]. Among them, *M*. *chrysogaster* and *M*. *leucogaster* were found in 1839 [25]. Then, *M*. *berezovskii* [26], *M*. *fuscus* [27], *M*. *anhuiensis* [28,29] and *M*. *cupreus* [30] were described successively in more than one century. However, it remains disputed precisely how many species exist in *Moschus*. One of the most extreme opinions insist that just one species exist in Moschidae [31,32]. However, most scholars believe that there are multiple species in *Moschus* [33,34]. In addition to the above opinions, there remain some differing and controversial points. For example, Sheng *et al*. (2007) believed that there were only three species (*M*. *moschiferus*, *M*. *berezovskii* and *M*. *chrysogaster*) that existed in this genus, and that *M*. *leucogaster* and *M*. *fuscus* should be considered a subspecies of *M*. *chrysogaster* (*M*. *c*. *leucogaster*, *M*. *c*. *fuscus*), and that *M*. *anhuiensis* should be regarded as a subspecies of *M*. *berezovskii* (*M*. *b*. *anhuiensis*). Among those species, the debate with regard *M*. *anhuiensis* is the most complex. In 1982, three unknown specimens of musk deer were found in Huoshan, Anhui province, China, and it was recognized as the subspecies (*M*. *m*. *anhuiensis*) of *M*. *moschiferus* based on morphological data [29]. Later, some researchers thought that it should be considered a subspecies (*M*. *b*. *anhuiensis*) of *M*. *berezovskii* rather than *M*. *moschiferus* and as based on the characters of fur texture, stripes and the structure of the skull [33,35,36,37,38]. However, further studies based on molecular phylogeny suggested that it should be recognized as a separate species [28,34].

For a long time, it was believed that northern Asia was the origin center of *Moschus* [21], in part because the most ancient fossils of *Moschus* were found in Tunggur, Inner Mongolia [40,41]. Accordingly, *M*. *moschiferus* was regarded as the most primitive species in existence for musk deer. In addition, this contention was supported by several molecular phylogenetic studies [34,42,43]. Su *et al*. combined the data of current distributions, fossil records and molecular data, and then concluded that the historical dispersion of musk deer might be from north to south China [43]. However, others consider *M*. *chrysogaster* as the most primitive musk deer [44], which is also supported by molecular phylogenetic studies [45]. The existence of discrepancy makes the origin and evolutionary history of *Moschus* as an additional interesting question for further investigation.

Mitochondrial DNA (MtDNA) markers are chosen frequently to study evolutionary history, biological identification, taxonomy, biogeography and phylogeny [46]. The use of mtDNAs can improve the probability of congruence between the mitochondrial genetic tree and the species tree, which is useful in resolving relationships between recently divergent taxa [47]. Notably, single-gene phylogenies often differ dramatically from studies involving multiple datasets, suggesting that they are often unreliable [48]. Thus, the complete mtDNA genome were gradually used to construct reliable phylogeny for determining evolutionary relationships among species or higher taxa with accurate timescales [49,50,51].

In the present study, we set out to examine sequence variation of the complete mtDNA genome, as well as mitochondrial genomic structure and organization from inclusive species of *Moschus* to address the following emergent issues: (i) species delimitation for controversial species based on a reliable tree with time-scales, and (ii) the evolutionary history and speciation events of *Moschus*. Whether geological events and environmental changes including uplift of the Qinghai Tibetan Plateau, climatic oscillations in Pleistocene have affected its speciation evolution, is one of the key determining objectives of the current study.

## Materials and Methods

### Ethical Statement

In the present study, collection of samples was performed within a long-term investigation project on *Moschus* and all samples were from individuals that died naturally and were found during field work. This investigation project and the sample collection were approved by the Forestry Administration. Our experimental procedures complied with the current laws on animal welfare and research in China, and were specifically approved by the Animal Research Ethics Committee of Anhui University.

### Specimen Collection, DNA Extraction, PCR Amplification, and Sequencing

Two samples of *M*. *anhuiensis* and two samples *M*. *chrysogaster* were collected from 2010 to 2013, and the detailed information was shown in S1 Table. Four complete mtDNA genome were obtained from above samples (KP684123, KP684124, KC425457, NC020017) [52,53]. Other sequences in the analysis were downloaded from the NCBI database (http://www.ncbi.nlm.nih.gov/pubmed/) (see S1 Table for a full list of sequences) [39,43,50,54,55,56,57,58].

We extracted total DNA using a standard proteinase K/phenol-chloroform protocol [59]. An EasyPure PCR Purification Kit (TransGene) was used to purify each DNA extraction. Twenty-one pairs of primers(S2 Table) were designed using Primer Premier 5.0 [60] based on *M*. *moschiferus* (NC013753) and *Rangifer tarandus* (AB245426). The product size of above primer pairs ranged from 635 bp to 1400 bp. Polymerase chain reaction (PCR) was performed using a reaction mixture (25 μL) containing 1 μL genomic DNA (concentration 10–50 ng/μL), 2.5 μL 10×buffer, 1 μL of 2.5 mM MgSO_4_, 2 μL of 2 mM dNTPs, 2.5 U *Taq* polymerase (Meridian Bioscience, Singapore) and 0.3 mM of each of the primers. Pure molecular biology grade water was added to reach the appropriate volume. The amplification protocol included an initial denaturation step of 95°C for 5 min; this was followed by 32 cycles of denaturation at 95°C for 30 s, primer annealing at 55°C for 30 s, and an extension at 72°C for 80 s, with a final extension at 72°C for 10 min. PCR products were purified using an EasyPure PCR Purification Kit (TransGene), and sequenced using previous primers and the BigDye Terminator v3.0 Ready Reaction Cycle Sequencing Kit (Applied Biosystems) following the manufacturer’s instructions on an ABI Prism 3730 automated sequencer. In addition, several different methods (e.g. BLAST search and translation test method) had been adopted to exclude potential nuclear mitochondrial pseudogenes [61].

### Sequence Analysis

Sequences were assembled by Seqman II (DNAStar, Madison, WI, USA) and checked by visual inspection to ensure the accuracy of variable sites identified by the program [62]. Protein-coding genes were identified by comparison with known complete mtDNA sequences of Ruminantia using Sequin 11.0. The 22 tRNA genes were identified using the software package tRNA Scan-SE 1.21 (http://lowelab.ucsc.edu/tRNAscan-SE 1.2.1). In addition, the DOGMA annotation software was used to check annotated genes [63]. All assembled and annotated *Moschus* mitochondrial genomes are available at GenBank, accession numbers are given in S1 Table. Moreover, A+T content was calculated using MEGA 5.05 [64]. Strand asymmetry was calculated using the formulae AT skew = [A−T]/[A+T] and GC skew = [G−C]/[G+C] [65], for the strand encoding the majority of the protein-coding genes. The complete alignment of nucleotides of the four *Moschus* mtDNAs was used to effect sliding window analyses using DnaSP v. 5 [66]. A sliding window of 300 bp and steps of 10 bp were used to estimate nucleotide diversity (*π*) for the entire alignment. Nucleotide diversity for the entire alignment was plotted against midpoint positions of each window, and gene boundaries as indicated. We calculated uncorrected genetic distances corrected by GTR + *I* + *G* for each mitochondrial gene separately, and did so using MEGA 5.05 [64].

Five different datasets were generated for different analyses. Dataset 1 composed of complete mtDNA, except the control region, from 14 species in Ruminantia, including four *Moschus* species, *M*. *chrysogaster*, *M*. *moschiferus*, *M*. *berezovskii* and *M*. *anhuiensis* (S1 Table). In Dataset 2, complete mtDNA, with the exception of the control region of the above defined four species in *Moschus* were included. In Dataset 3, 12S rRNA and Cyt *b* gene sequences of six species in *Moschus* (*M*. *leucogaster*, *M*. *fuscus*, *M*. *chrysogaster*, *M*. *moschiferus*, *M*. *berezovskii* and *M*. *anhuiensis*) were included. Dataset 4 composed of 13 mitochondrial protein gene in Ruminantia, including four *Moschus* species, *M*. *chrysogaster*, *M*. *moschiferus*, *M*. *berezovskii* and *M*. *anhuiensis* (S1 Table). In Dataset 5, all mitochondrial protein gene were included, except ND6 gene (S1 Table). We selected the best fit model of evolution for these datasets using MrModeltest 1.0 b [67] based on the AIC criterion.

### Phylogenetic Analyses

**Fig 2:**
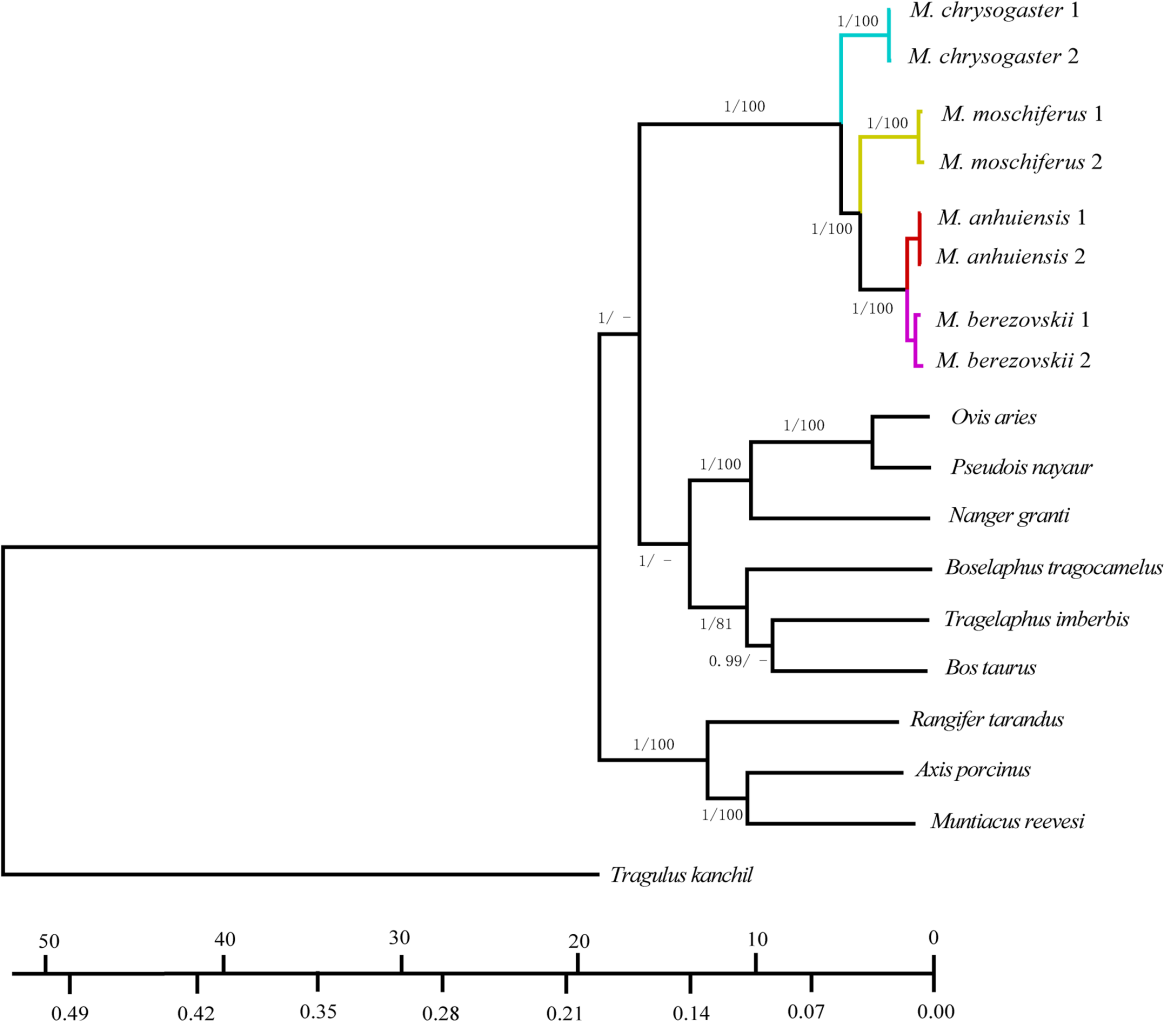
Phylogram showing the phylogenetic relationship in *Moschus*. The species from *Moschus* were highlight in different colors. The values on nodes indicate Bayesian posterior probabilities and ML support; “-” indicated that the value was less than 70.

**Fig 3:**
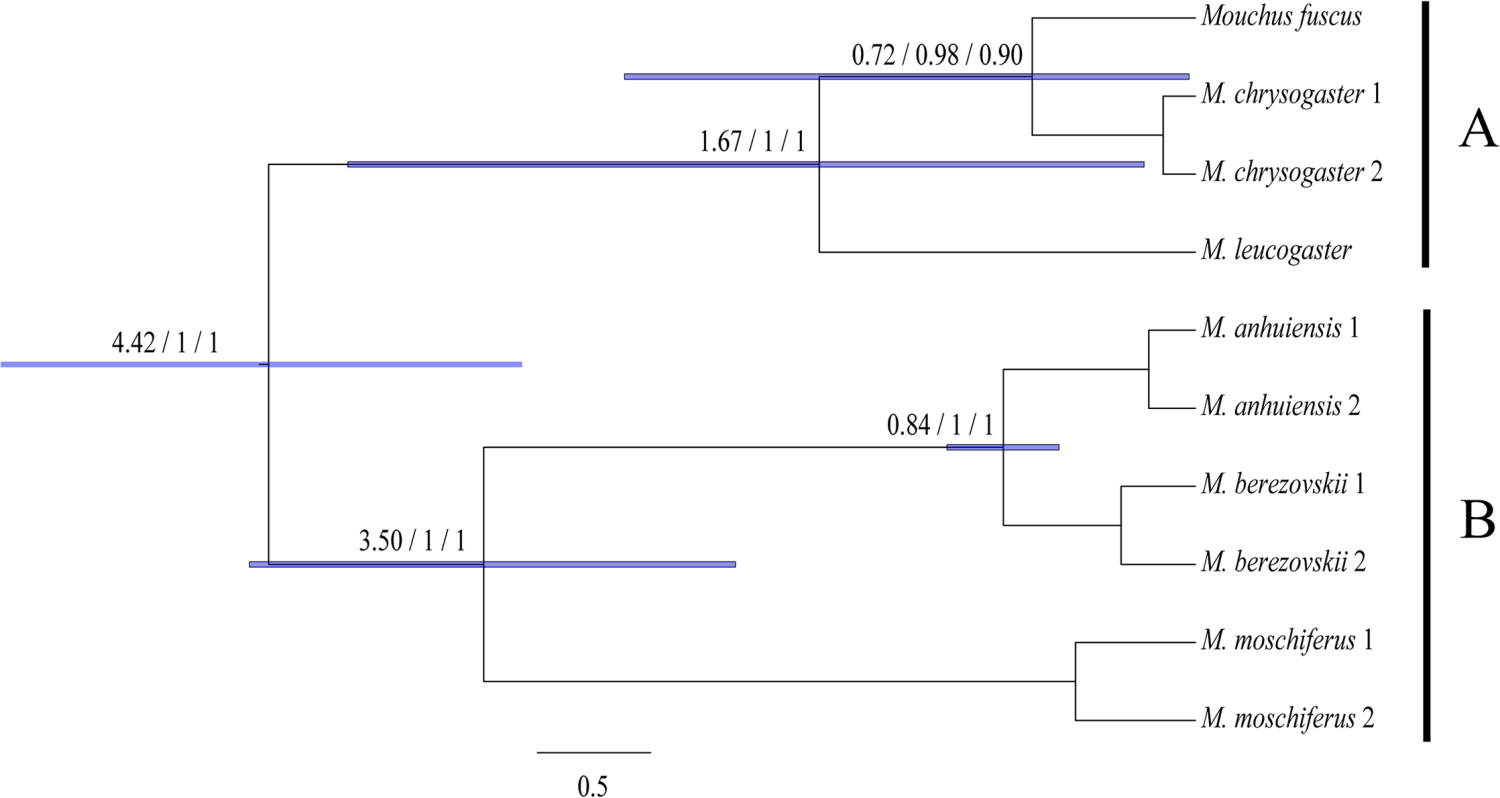
Phylogram showing the phylogenetic relationship in Moschidae. The values on nodes include three parts. The first two values indicate the split time and Bayesian posterior probabilities which were calculated by BEAST 1.7.4. The last values were the Bayesian posterior probabilities calculated by MrBayes 3.1.2.

Eighteen species in the Ruminant (Dataset 1) group were used to reconstruct a phylogenetic study using Maximum Likelihood (ML) and Bayesian methods, with *Tragulus kanchil* used as an outgroup (Fig 2)[50]. Before reconstructing the phylogenetic trees, sequence alignment was carried out using Clustal X 1.8 software [68], followed by manual adjustment. All ambiguous regions, i.e., those that involved ambiguity for the gap positions, were excluded from the analyses to avoid erroneous hypotheses of primary homology. ML analyses were performed in RAxML version 8 [69] and a general time reversible model of nucleotide substitution under the Gamma model of rate heterogeneity (i.e., GTRCAT). Support for internal branches for the best-scoring tree was evaluated via the bootstrap test with 1000 iterations. Bayesian inference of phylogeny was performed using the MrBayes 3.1.2 software program (http://mrbayes.csit.fsu.edu/index.php) [70], with the same best fit substitution model used as that selected for the ML analysis. MrBayes analyses simultaneously initiate two Markov Chain Monte Carlo (MCMC) model runs to provide additional confirmation of convergence of posterior probability distributions. Analyses were run for 10,000,000 generations. Chains were sampled every 1000 generations. When the average standard deviation of split frequencies reached a value less than 0.01. The first 10% of the total trees were discarded as ‘‘burn-in” and the remaining trees were used to calculate Bayesian posterior probabilities. MrBayes analyses were also implemented for Dataset 3, Dataset 4 and Dataset 5 with the GTR + *I* model (Fig 3 and S1 Fig). We assumed that the tree topology that was derived from analyses of the entire mitochondrial sequence was the true mitochondrial gene tree. To determine the support provided by each gene or region to this topology, we ran ML, which was based on the bootstrap test with 1000 iterations on each gene or region separately in MEGA 5.05(S2 Fig) [64]. In addition, to obtain the estimated best fit evolution model for each mitochondrial region, we performed analyses separately as described above using the MrModeltest 1.0 b software program [67] in Paup*4.0b10 [71], which was based on the AIC criterion.

### Species Delimitation

**Fig 4:**
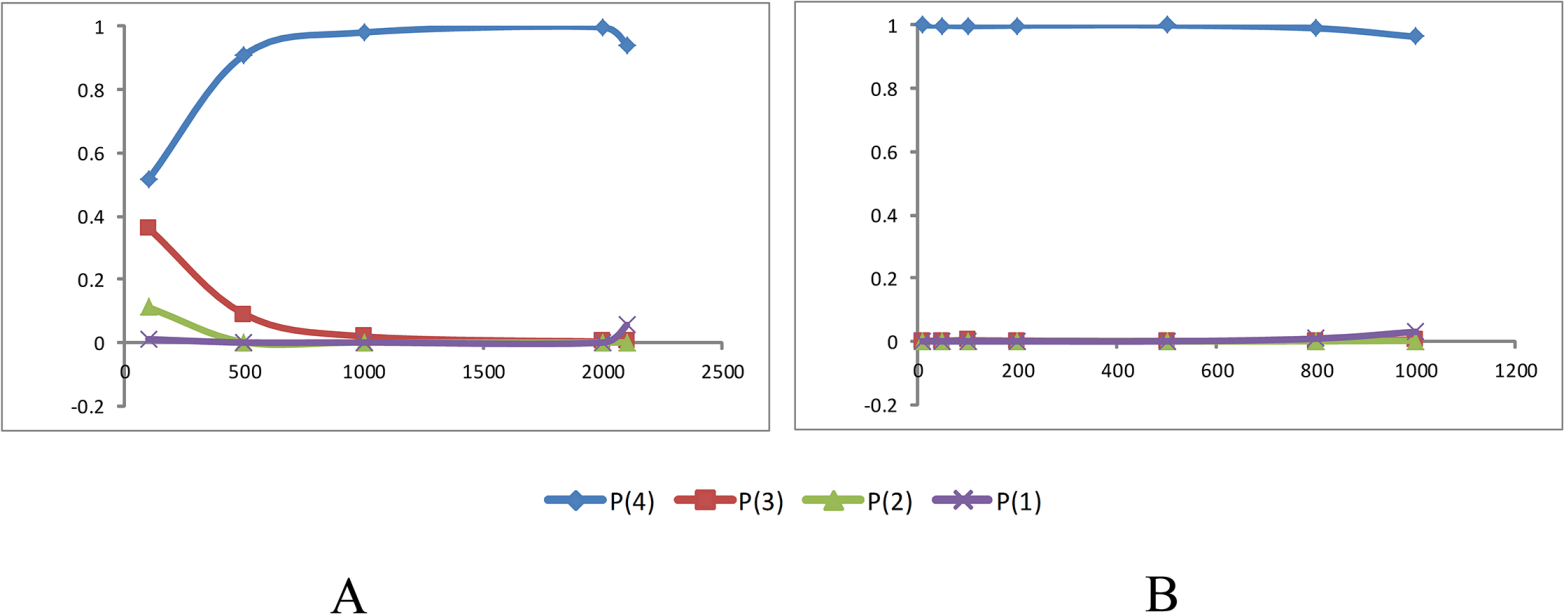
The species delimitation results based on the complete mt genome in four species of *Moschus* (*M*. *chrysogaster*, *M*. *moschiferus*, *M*. *berezovskii* and *M*. *anhuiensis*). A: the posterior probability of number of species when θ changed (τ_0_ = 1: 100); B: the posterior probability of number of species when τ_0_ changed (θ = 1: 2 000). P(4) means that the probability of four species; P(3) means that the probability of three species; P(2) means that the probability of two species; P(1) means that the probability of one species.

The species delimitation in *Moschus* was implemented in BPP v. 3.0 [72,73] based on reversible-jump Markov Chain Monte Carlo (rjMCMC) model sampling in Dataset 2. The guide tree was collected from the phylogenetic analyses. We assessed the impact of ancestral effective population size and time of divergence on species delimitation by testing a range of different prior distributions for θ and τ_0_. First, we fixed the τ_0_ (1: 100), and θ acted as the variable. Low values for priors (i.e., at a frequency of 1:10) generally infer a large population sizes and deep divergence, whereas higher values infer small population sizes and shallow divergence for θ and τ_0_, respectively. Secondly, we fixed θ (i.e., at a ratio of 1:2000), and τ acted as the variable. Based on the results (Fig 4), we fixed θ (1:2000) and τ (1:10) for the prior distributions (Table 1). Each analysis ran three independent chains of 500 000 steps, sampling every fifth step, with 100 000 burn-in steps. In addition, for Dataset 3, we included two other species, *M*. *leucogaster* and *M*. *fuscus* to probe whether both of these species are independent species. The prior distributions for θ and τ in the context of this analyses were identical to that described for the first part of the analysis (Table 1).

**Table 1:** The species delimitation results based on the complete mt genome in *Moschus*. The prior distributions were fixed on θ (1: 2000) and τ (1: 10).

											Tree			
	P(6)	P(5)	P(4)	P(3)	P(Mf)	P(Ml)	P(Mc)	P(Mm)	P(Mb)	P(Ma)	Tree 1	Tree 2	Tree 3	Tree 4
Dataset 2	-		1.00	0.00	-	-	1.00	1.00	1.00	1.00	0.69	0.22	0.09	0.00
Dataset 3	0.95	0.05	0.00	-	0.95	1.00	0.96	1.00	1.00	1.00	0.54	0.28	0.13	0.03

**Note: (1)** Ml, Mf, Mc, Mm, Mb, Ma represents *M*. *leucogaster*, *M*. *fuscus*, *M*. *chrysogaster*, *M*. *moschiferus*, *M*. *berezovskii* and *M*. *anhuiensis*, respectively. **(2)** “Tree1”, “Tree 2”, “Tree 3”, “Tree 4”represents “(Mc, (Mm, (Mb, Ma))) or ((Ml, (Mc, Mf)), (Mm, (Mb, Ma)))”, “(Mm, (Mc, (Mb, Ma))) or (Mm, ((Ml, (Mc, Mf)), (Mb, Ma)))”, “((Mm, Mc), (Mb, Ma)) or ((Mm, (Ml, (Mc, Mf))), (Mb, Ma))”, “(Mc, (MbMa, Mm)) or ((McMf, Ml), (Mm, (Mb, Ma)))”, respectively.

### Divergence Time Analyses

To estimate divergence times in *Moschus*, we applied a Bayesian MCMC method (Dataset 1) based on mitochondrial genomes, which employs a relaxed molecular clock approach, as implemented in BEAST 1.7.4 [74]. We assumed a relaxed uncorrelated log normal model of lineage variation and a Yule Process prior to the branching rates based on the HKY+I +G model, and as recommended by MrModel test 1.0 b [67]. Four replicates were run for 10,000,000 generations with tree and parameter sampling that occurred every 1,000 generations for the first 10% of samples that were discarded as burn-in. All parameters were assessed by visual inspection using Tracer v. 1.5 [75]. The tree was generated and visualized with TreeAnnotator v. 1.6.1 [76] and FigTree v. 1.3.1 [77], respectively. All calibration points were derived from Hassanin *et al*. (S3 Table) [50]. In addition, more species (for example adding *M*. *leucogaster* and *M*. *fuscus*) with relatively shorter sequences (i.e., containing 12S and Cyt *b*, and contained in Dataset 3) were also used to estimate divergence times in *Moschus*, and were based on the calibration points of the analysis described above. Relaxed uncorrelated log normal models of lineage variation and the Yule Process were set by basing them on the GTR + I model as recommended by MrModeltest 1.0 b [67].

## Results

### Genomic Organization and Gene Arrangement

The complete mtDNA sequence of *Moschus* ranged from 16351 to 16354, and contained 13 protein-coding genes (i.e., ATP6, ATP8, COI, COII, COIII, ND1, ND2, ND3, ND4, ND5, ND6, ND4L, and Cyt *b*), two rRNAs (i.e., 12S rRNA and 16S rRNA), 22 tRNAs and a control region. The base composition of mtDNAs was A (29.6%), G (14.8%), C (32.8%), and T (22.9%) (S3 Fig, S4 Table), and the percentage of A and T (52.5%) was slightly higher than that of G and C. The heavy DNA strand (H-strand) carried most of the genes; i.e., 12 protein-coding genes, two rRNAs, and 14 tRNAs. While ND6 and eight tRNAs were located on the L-strand. The arrangement of the whole mitochondrial genome of *Moschus* matched known typical mammalian patterns [78]. The total length of the 13 protein-coding genes was 11,315 bp, which represented about 69.2% of the entire mitochondrial genome in *Moschus*.

The longest gene was ND5 (1,821 bp), which was located between tRNA^Leu^ (CUN) and ND6, and the shortest was ATP8 (201 bp), which was located between tRNA^Lys^ and ATP6. Eleven of the 13 protein-encoding genes ended with a complete (TAA) or an incomplete (T or TA) stop codon; the latter was presumably completed as TAA by post-transcriptional polyadenylation [79,80]. However, Cyt *b* ends with AGA, and ND2 ends with AGA. Ten of the protein-coding genes started with an ATG codon and ND2 and ND3 started with an ATA codon. Notably, ND5 in *Moschus* began with ATT while all other species in our study displayed ATA as the start codon.

**Fig 5:**
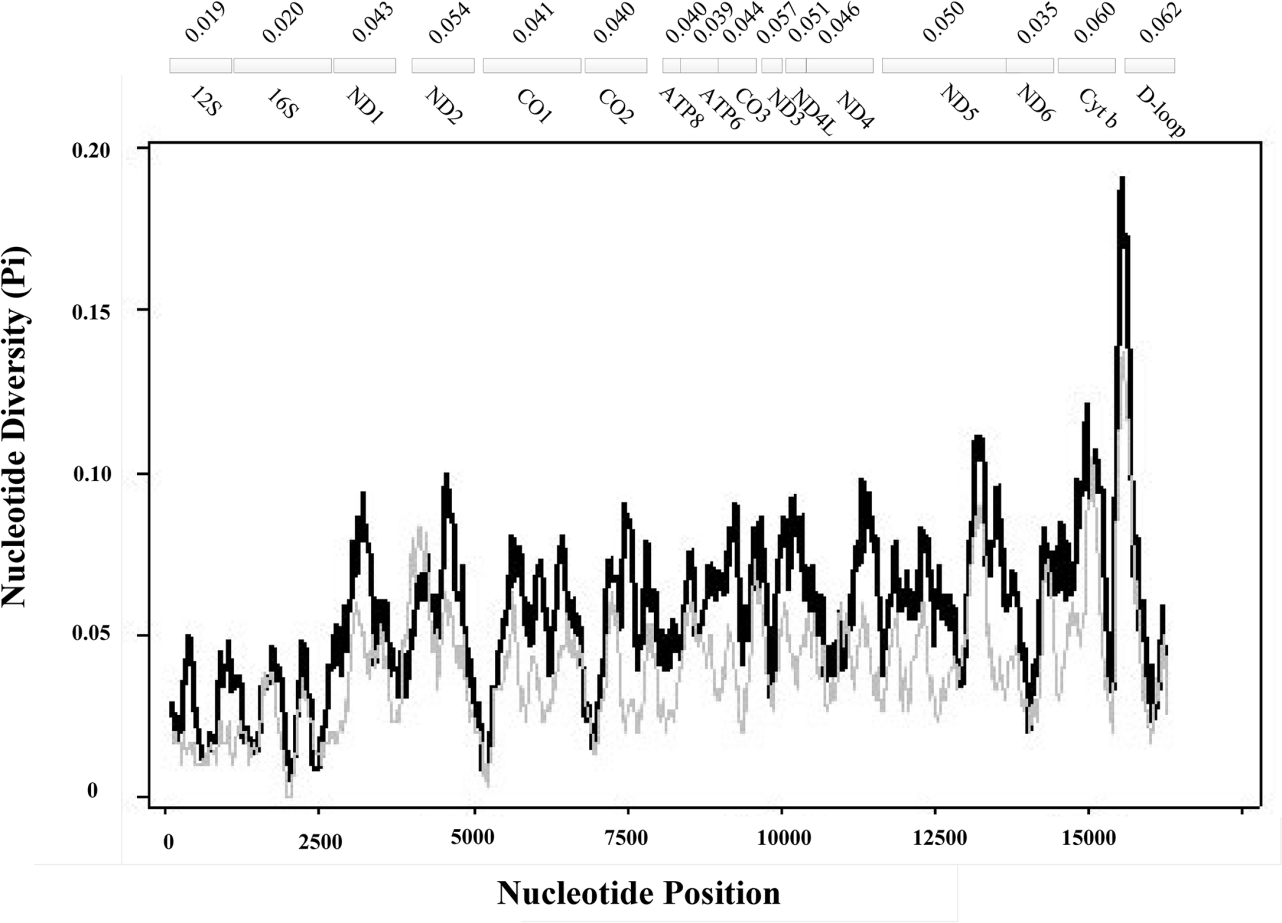
Sliding window analyses showing the nucleotide diversity based on alignment of complete mtDNAs of four species in *Moschus* (*M*. *chrysogaster*, *M*. *moschiferus*, *M*. *berezovskii* and *M*. *anhuiensis*). The black line shows the value of nucleotide diversity (*π*) in a sliding window analysis of window size 300 bp with step size 10, the value is inserted at its mid-point. Gene boundaries are indicated with an indication of the total number of variable positions per gene; ATP8 with ATP6, ND4L with ND4, and ND5with ND6 are overlapping.

Sliding window analysis of the nucleotide alignment of four species in *Moschus* provided an indication of nucleotide diversity (π) within and between mitochondrial genes, which revealed significant regional variation across the alignment (Fig 5). The plot readily showed a high degree of nucleotide variation within and between genes amongst the aligned *Moschus* genomes for any given window of 300 bp and steps of 10 bp, with the Pi value ranging from 0.007 to 0.119. Coupled with computation of the number of variable positions per unit length of a given gene, the sliding window showed that the genes with relatively low sequence variability included the 12S rRNA gene (0.019), and the 16S rRNA gene (0.020), while the genes with high sequence variability included Cyt *b* (0.060) and the Control region (0.062).

**Fig 6:**
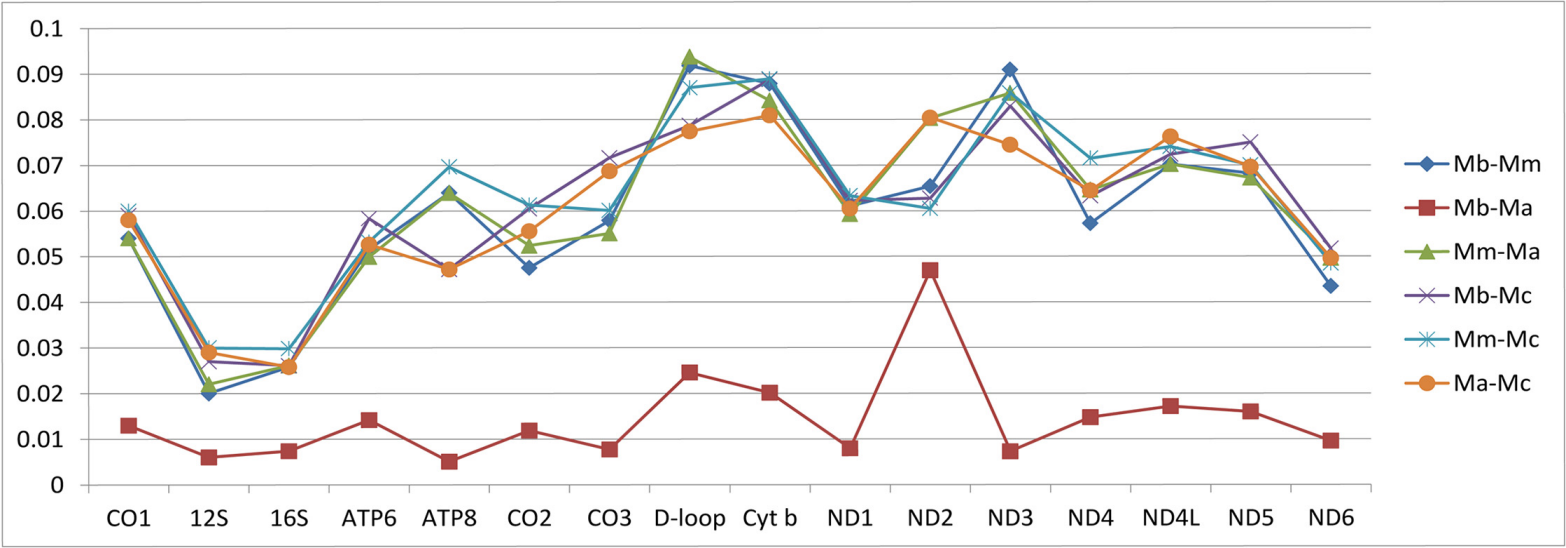
The genetic distance among four species of *Moschus* based on each gene in mitochondrial genome. Ma, Mb, Mm, Mc represents *M*. *anhuiensis*, *M*. *berezovskii*, *M*. *moschiferus* and *M*. *chrysogaster*, respectively.

Overall mean distances of each mitochondrial gene based on the bootstrap method in the Kimura 2-parameter model showed a lack of uniformity. For example, for the Control region, Cyt *b* and ND3 nearly obtained a doubled value as compared with that of the 12S rRNA and 16S rRNA. This phenomenon is also supported by the relative evolution rate that was calculated by Beast (S4 Fig) and the estimated model parameters (S4 Table). Uncorrected genetic distances among four species ranged from 1.4% (*M*. *berezovskii*—*M*. *anhuiensis*) to 4.8% (*M*. *chrysogaster*- *M*. *berezovskii*) (Fig 6).

### Phylogenetic Reconstructions

Topologies recovered from the maximum likelihood (ML) and Bayesian inference (BI) analyses of complete mtDNA for 18 Ruminant species (Dataset 1) were highly congruent, with only slight differences found in the bootstrap support or posterior probability values for most nodes (Fig 2). The 17 species were divided into three major lineages in which each represented Cervidae, Bovidae, and Moschidae. The lineage of Bovidae is a sister group to Moschidae. In Moschidae, there are four clades for which there is a high level of Bayesian posterior probabilities and ML support. Each clade represents one species. *M*. *chrysogaster* is at the base of the tree and *M*. *berezovskii* and *M*. *anhuiensis* are sister groups of the inner clade (Fig 2). In addition, the phylogenetic analyses based on mitochondrial gene have similar topologies (S1 and S2 Figs).

Topologies recovered from Bayesian inference (BI) of 12S and Cyt *b* for six *Moschus* species (Dataset 3) obtained highly posterior probability values for most nodes (Fig 3). Those six species were divided into two major lineages: *M*. *leucogaster*, *M*. *fuscus* and *M*. *chrysogaster* that were clustered into one lineage (A) and the other three species that were clustered into the other lineage (B) (Fig 3). In lineage A, *M*. *leucogaster* was at the base position of the phylogenetic tree, and *M*. *berezovskii* and *M*. *anhuiensis* were sister groups in the inner clade of lineage B.

The molecular dating of Dataset 1 also showed estimated divergence times of four species present in the Moschidae family. The time since the most recent common ancestor (MRCA) of *Moschus* was estimated as 4.42 Ma (i.e., 95% CI, 3.40–5.51 Ma). The MRCA of *M*. *moschiferus*, *M*. *berezovskii* and *M*. *anhuiensis* was 3.50 Ma (i.e., 95% CI, 2.53–4.44 Ma), and the split between *M*. *berezovskii* and *M*. *anhuiensis* was estimated at 0.84 Ma (i.e., 95% CI, 0.63–1.05) (Fig 2). The molecular time dating results of Dataset 3 showed that the time since MRCA of *M*. *leucogaster*, *M*. *fuscus*, and *M*. *chrysogaster* was 2.01 Ma (i.e., 95% CI, 0.33–4.09 Ma), and the split between *M*. *fuscus* and *M*. *chrysogaster* was estimated at 0.95 Ma (i.e., 95% CI, 0.02–2.40 Ma) (Fig 3).

### Species Delimitation

The species delimitation based on Dataset 2 (*M*. *chrysogaster*, *M*. *moschiferus*, *M*. *berezovskii* and *M*. *anhuiensis*) showed that the probability values of four separate species exceeded 0.95, which demonstrated that the four species could be regarded as valid species. The topology [i.e., (Mc, (Mm, (Mb, Ma)))] attained an approval probability greater than 0.69, while the other three kinds of topologies were less than 0.25. Further, the six species can be regarded as valid species in the species delimitation of Dataset 3 because of the probability values of six separate species higher than 0.95. The topology, ((Ml, (Mf, Mc)), (Mm, (Mb, Ma))), attained an approval probability of 0.54. Moreover, the other three kinds of topology had a probability of less than 0.25. These two highly approved topologies were similar to the above phylogenetic study (Figs 2 and 3).

## Discussion

### Mitochondrial Genome Annotation and Features

The mtDNA genome of four species in *Moschus* is nearly identical to those of other ruminants in many respects, in which there are no introns, no long intergenic spacers, and only a few overlapping sequences [80,81,82]. The overall mean base composition was: A, 34.0%; C, 25.0%; G, 12.9%; and T, 28.1%. The A + T content (62.1%) was higher than the C + G content (37.9%), which indicated a strong AT bias, which was similar to other ruminants in our studies (58.8–63.7%). Guanine (G) is the rarest nucleotide; the percentage of the other three bases were roughly equal to each other (S4 Table), similar to other vertebrate animals [78,80,83].

GC and AT skews are a measure of compositional asymmetry. In amniote mtDNA, GC-skew values are all negative (G<C), while AT-skews are positive (A>T) [65]. In mtDNA of *Moschus*, the GC-skew (−0.3175 to −0.3245) and the AT-skew (0.0915–0.0968) values were in accord with this principle (S4 Table). In sliding window analysis, the largest value in the curve was 0.129 (Pi) and the largest value of the number of variable positions per unit length of the gene was CR (0.062).

When we compared the 11 ruminant species from four families with respect to the predicted initiation and termination codons of 13 mitochondrial protein-coding genes (Table 2), we found that most protein-coding genes used ATG as the start codon, and only a few species started with the GTG, ATT or ATA sequences. Stop codons were also similar across different species with TAA, TA- and T- occurring most frequently. Notably, ND5 in *Moschus* showed a differential pattern, which started with ATT and ended with TAA. Others in the Artiodactyla, started with ATA and ended with TAA. Based on our phylogenetic study, we inferred that the mutation (synonymous transition) had occurred in the *Moschus* ancestor.

**Table 2:** Predicted initiation and termination codons for 13 mitochondrial protein-coding genes in 11 species in Artiodactyla.

Gene	Predicted initiation and termination										
	A	B	C	D	E	F	G	H	I	J	K
CO1	ATG/TAA	ATG/TAA	ATG/TAA	ATG/TAA	ATG/TAG	ATG/TAA	ATG/TAA	ATG/TAA	ATG/TAA	ATG/TAA	ATG/TAA
ATP6	ATG/TAA	ATG/TAA	ATG/TAA	ATG/TAA	ATG/TAA	ATG/TAA	ATG/TAA	ATG/TAA	ATG/TAA	ATG/TAA	ATG/TAA
ATP8	ATG/TAA	ATG/TAA	ATG/TAA	ATG/TAA	ATG/T	ATG/TAA	ATG/TAA	ATG/TAA	ATG/TAA	ATG/TAA	ATG/TAA
CO2	ATG/TAA	ATG/TAA	ATG/TAA	ATG/TAA	ATG/TAG	ATG/TAA	ATG/TAA	ATG/TAA	ATG/TAA	ATG/TAA	ATG/TAA
CO3	ATG/TA	ATG/TA	ATG/TA	ATG/TA	ATG/TA	ATG/TA	ATG/T	ATG/TA	ATG/TA	ATG/T	ATG/TA
Cyt b	ATG/AGA	ATG/AGA	ATG/AGA	ATG/AGA	ATG/AGA	ATG/TAA	ATG/AGA	ATG/AGA	ATG/AGA	ATG/AGA	ATG/AGA
ND1	ATG/TAA	ATG/TAA	ATG/TAA	ATG/TAA	ATG/TAA	ATG/TAA	ATG/TAA	ATG/TAA	ATG/TAA	ATG/TAA	ATG/TAA
ND2	ATA/TAG	ATA/TAG	ATA/TAG	ATA/TAG	ATA/TAG	ATA/TAG	ATA/TAG	ATA/TAG	ATA/TAG	ATA/TAG	ATA/TAG
ND3	ATA/TA	ATA/TA	ATA/TA	ATA/TA	ATA/TA	ATA/TA	ATA/TA	ATA/TA	ATA/TA	ATA/TA	ATA/TA
ND4	ATG/T	ATG/T	ATG/T	ATG/T	ATG/T	ATG/T	ATG/T	ATG/T	ATG/T	ATG/T	ATG/T
ND4L	ATG/TAA	ATG/TAA	ATG/TAA	ATG/TAA	GTG/TAA	GTG/TAA	ATG/TAA	ATG/TAA	ATG/TAA	ATG/TAA	ATG/TAA
ND5	ATT/TAA	ATT/TAA	ATT/TAA	ATT/TAA	ATA/TAA	ATA/TAA	ATA/TAA	ATA/TAA	ATA/TAA	ATA/TAA	ATA/TAA
ND6	ATG/TAA	ATG/TAA	ATG/TAA	ATG/TAA	ATG/TAA	ATG/TAA	ATG/TAA	ATG/TAA	ATG/TAA	ATG/TAA	ATG/TAA

Notes: A: *M*. *berezovskii* (NC 012694, JQ409122), B: *M*. *moschiferus* (NC 013753, JN632662), C: *M*. *anhuiensis* (NC 020017, KP684124), D: *M*. *chrysogaster* (KC 425457, KP684123), E: *Tragulus kanchil* (NC 020753), F: *Rangifer tarandus* (NC 007703), G: *Muntiacus reeves* (NC 004069), H: *Bos taurus taurus* (EU 177832), I: *Nanger granti* (NC 020725), J: *Ovis aries* (NC 001941), K: *Axis porcinus* (NC 020681).

### Species Delimitation

Although seven potential *Moschus* species were not all included in this study, our results still obtained reliable information of species delimitation in *Moschus*. The six species (i.e., *M*. *leucogaster*, *M*. *fuscus*, *M*. *chrysogaster*, *M*. *moschiferus*, *M*. *berezovskii* and *M*. *anhuiensis*) in the present study were all recognized as a separate species (Figs 2 and 3., Table 1). Therefore, multiple species exist in *Moschus* [33] rather than only one species [31,32]. Among the six species analyzed in our study, the taxonomic status of *M*. *anhuiensis* was the most controversial. It was considered as a sub-species of *M*. *moschiferus* [29] or *M*. *berezovskii* [33,35,38]. In 1999, Li *et al*. set it as a valid species based on the Cyt *b* sequence (367 bp) and morphological variation [28]. Further, the later phylogenetic study also supported it as a separate species, and was considered the sister group of *M*. *chrysogaster* and *M*. *berezovskii* [34]. Our study supported the notion that *M*. *anhuiensis* was a valid species (Table 1), and it was the sister group of *M*. *berezovskii* (Figs 2 and 3). This result could be supported by the discontinuity of the distribution area between *M*. *berezovskii* and *M*. *anhuiensis* (Fig 1).

### Phylogenetic Relationship and Origin of Present Musk Deer

Historically, *M*. *moschiferus* was regarded as the primitive species in *Moschus*. Vislobokova *et al*. proposed that Moschidae originated from the north of Asia, since *M*. *grandaevus* and *M*. *primaevus* were collected from Oligocene formations in Mongolia, and were regarded as the most primitive fossil in *Moschus* [21]. Presently, only *M*. *moschiferus* is distributed through the north of Asia, thus it was regarded as the most primitive musk deer [21]. This viewpoint was strengthened by some additional molecular phylogenetic studies [34,42,43]. However, other investigators in the field contended that *M*. *chrysogaster* was more primitive than *M*. *moschiferus* based on morphological or molecular data [44,45]. In this study, two main evolutionary lineages (A, B) were disclosed in *Moschus* (Figs 2 and 3). One of them, was referred to as lineage A, which contained *M*. *leucogaster*, *M*. *fuscus* and *M*. *chrysogaster*, and were distributed in the Tibetan Plateau margin (Fig 1). Moreover, lineage B was composed of *M*. *moschiferus*, *M*. *berezovskii* and *M*. *anhuiensis*, which were distributed in the area that was located around the Sichuan basin, Qinling and the adjacent areas, as well as the Dabie mountain, east Asia and the far east area (Fig 1). However, in our study, *M*. *moschiferus* failed to occupy the most basal branches of the phylogenetic tree (Figs 2 and 3). Therefore, *M*. *moschiferus* was not the most primitive musk deer.

Molecular dating very clearly showed that speciation events of *Moschus* occurred during 4.42 Ma to 0.84 Ma (Figs 2 and 3). According to the topology, lineage A was distributed in the Tibetan Plateau margin, branched off from the most common ancestor of musk deer (about 4.42 Ma ago) and then followed the bifurcation forming the *M*. *moschiferus* lineage and the lineage that clustered *M*. *berezovskii* and *M*. *anhuiensis* (i.e., around 3.50 Ma before present). The remaining speciation events happened rather recently (i.e., less than 2.0 Ma). During 8 Mya—2.6 Mya, the average altitude of the Tibetan Plateau was raised by approximately 3,000 m, and was associated with high mountains and deep valleys that were generated in a way that separated several major rivers that ran in parallel [3,4]. The uplift of the Tibetan Plateau obstructed the northward flow of warm, wet-air from India across the mainland [8,9,10]. In addition, the Ice Age began about 2.8 million years ago [11]. Some studies have shown that ecological speciation events were profoundly accelerated by those geographic barriers, climatic changes and alternating glacial-interglacial periods [5,6,7,12,13]. In brief, our results suggested that the variance patterns of genetic structures of Moschidae may have resulted from: (1) the uplift of the Tibetan Plateau followed by increased aridification, and desertification in northern China in the Middle Pleistocene age, (2) the monsoon and the existence of Qinling Mountains and Liupan Mountains, and (3) the glacial cycles from the late Middle Pleistocene to the early late Pleistocene age.

Previously, Deng *et al*. proposed that the Tibet Plateau may represent a primitive form area for some Megaherbivores and large predators like *Pseudois nayaur* (Bovidae, Artiodactyla), *Coeloonta thibetana* (Rhinocerotidae, Perissodactyla), *Equus kiang* (Equidae, Perissodactyla), *Panthera uncia* (Felidae, Carnivora) [11]. Therefore, on the basis of the above discussion, we contended that the ancestor of the present musk deer might have originated from the Tibetan Plateau. Combining the data of current distributions and phylogenetic results of this study (Figs 1, 2 and 3), we suggested that the most direct ancestor of present musk deers were distributed in the Tibet Plateau margin or adjacent mountains that are located around the Sichuan basin.

During the past 5 million years, the orogenic movement and climate change prompted its divergence and speciation in *Moschus*. Lineage A, which is composed of *M*. *leucogaster*, *M*. *fuscus* and *M*. *chrysogaster*, represented the clade that inhabited the forested marginal area of the Tibet Plateau, and occupying land from Kashmir to Qinghai, China. Lineage B represented the clade that expanded to the far east (*M*. *moschiferus*), as well as the mountains that are located around the Sichuan Basin (*M*. *berezovskii*) and the Dabie mountain (*M*. *anhuiensis*). Although earliest musk deer fossils of the Genus *Moschus* (i.e., *M*. *grandaevus*, *M*. *primaevus*) were found in the north of Asia [21], we believe that they may not be the most direct ancestor of the present musk deer.

## Supporting Information

S1 Data Alignments for eighteen complete mitochondrial genome in the Ruminant (Dataset 1). (TXT)

S2 Data Alignments for complete mtDNA, with the exception of the control region of the above defined four species in *Moschus* were included (Dataset 2). (TXT)

S3 Data Alignments of 12S rRNA and Cyt b gene sequences of six species in *Moschus* (*M*. *leucogaster*, *M*. *fuscus*, *M*. *chrysogaster*, *M*. *moschiferus*, *M*. *berezovskii* and *M*. *anhuiensis*) were included (Dataset 3). (TXT)

S4 Data Alignments of 13 mitochondrial protein gene in Ruminantia, including four *Moschus* species, *M*. *chrysogaster*, *M*. *moschiferus*, *M*. *berezovskii* and *M*. *anhuiensis* (Dataset 4). (TXT)

S5 Data Alignments of all mitochondrial protein gene were included, except ND6 gene, in Ruminntia (Dataset 5). (TXT)

S1 Fig Phylogram showing the phylogenetic relationship in ruminant which were calculated by MrBayes 3.1.2. The values on nodes indicate Bayesian posterior probabilities. A. The phylogenetic relationship in *Moschus* based on 13 protein-coding gene. B. The phylogenetic relationship in *Moschus* based on 13 protein-coding gene except ND6 gene.
(TIF)

S2 Fig Phylogram showing the phylogenetic relationship in *Moschus* which were calculated by Maximum likelihood in Mega 5.05. The values on nodes indicate Bootstrap support.
(TIF)

S3 Fig Diagram showing the G/C content of eleven species mtDNA genomes in ruminant. (TIF)

S4 Fig The relative evolution rate of each gene in mitochondrial genome of *Mochus*. The relative evolution rate of CO1 was regard as the standard control value.
(TIF)

S1 Table Information on the studied species including mtDNA genome length along with accession numbers for GenBank. (DOC)

S2 Table Overlap PCR primer pairs of the mitochondrial DNA in *Moschus*. (DOC)

S3 Table Calibration points used for divergence time estimates within Cetartiodactyla. (DOCX)

S4 Table Comparison of the model of evolution used in Bayesian analyses, overall base frequencies, AT skew, and GC skew. Model of evolution was calculated based on the gene in *Mouchus*.
(DOCX)
