# Supplementary figures and images for: Species Delimitation in the Genus Moschus (Ruminantia: Moschidae) and Its High-Plateau Origin
Source: PLoS One. 2015 Aug 17;10(8):e0134183. doi: 10.1371/journal.pone.0134183 (PMC4539215; doi:10.1371/journal.pone.0134183)

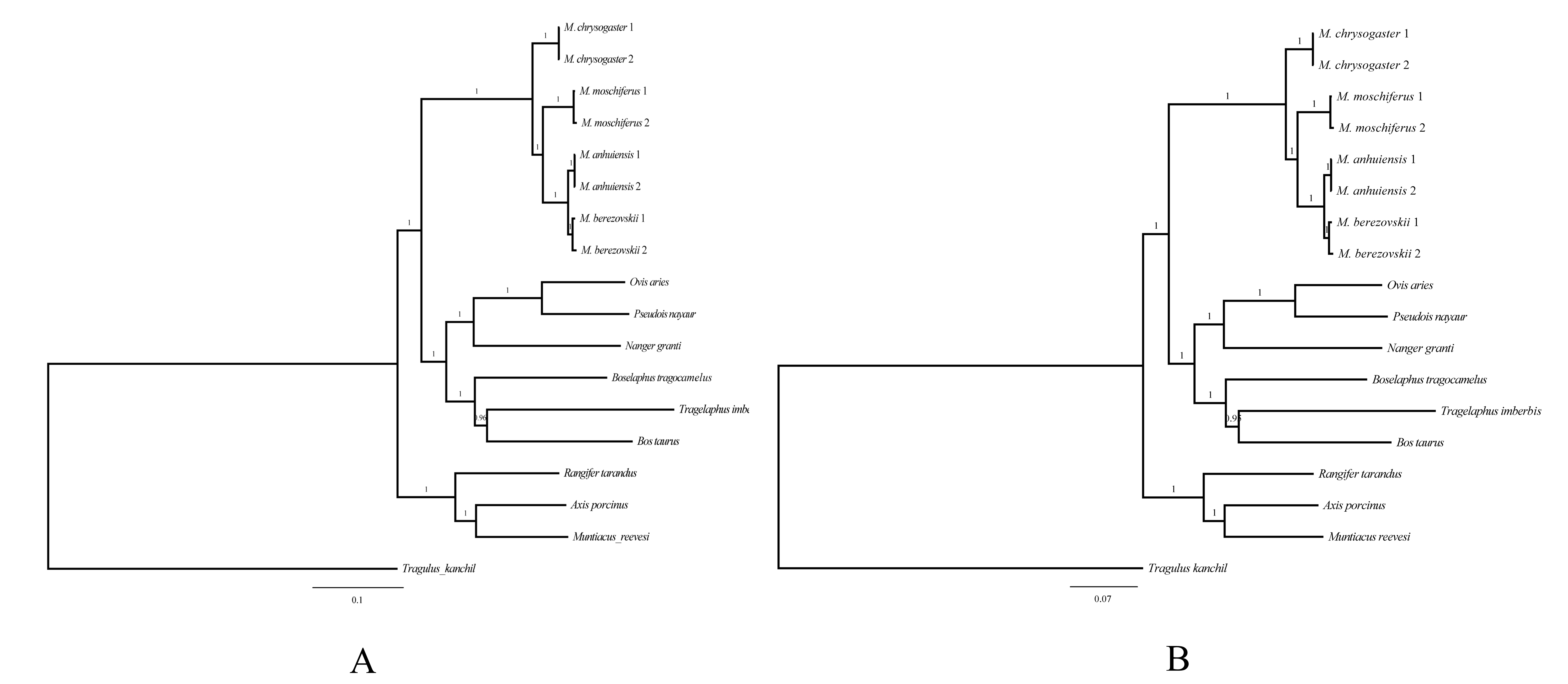

Supplement: S1 Fig — The values on nodes indicate Bayesian posterior probabilities. A. The phylogenetic relationship in Moschus based on 13 protein-coding gene. B. The phylogenetic relationship in Moschus based on 13 protein-coding gene except ND6 gene. (TIF) [file pone.0134183.s006.tif]

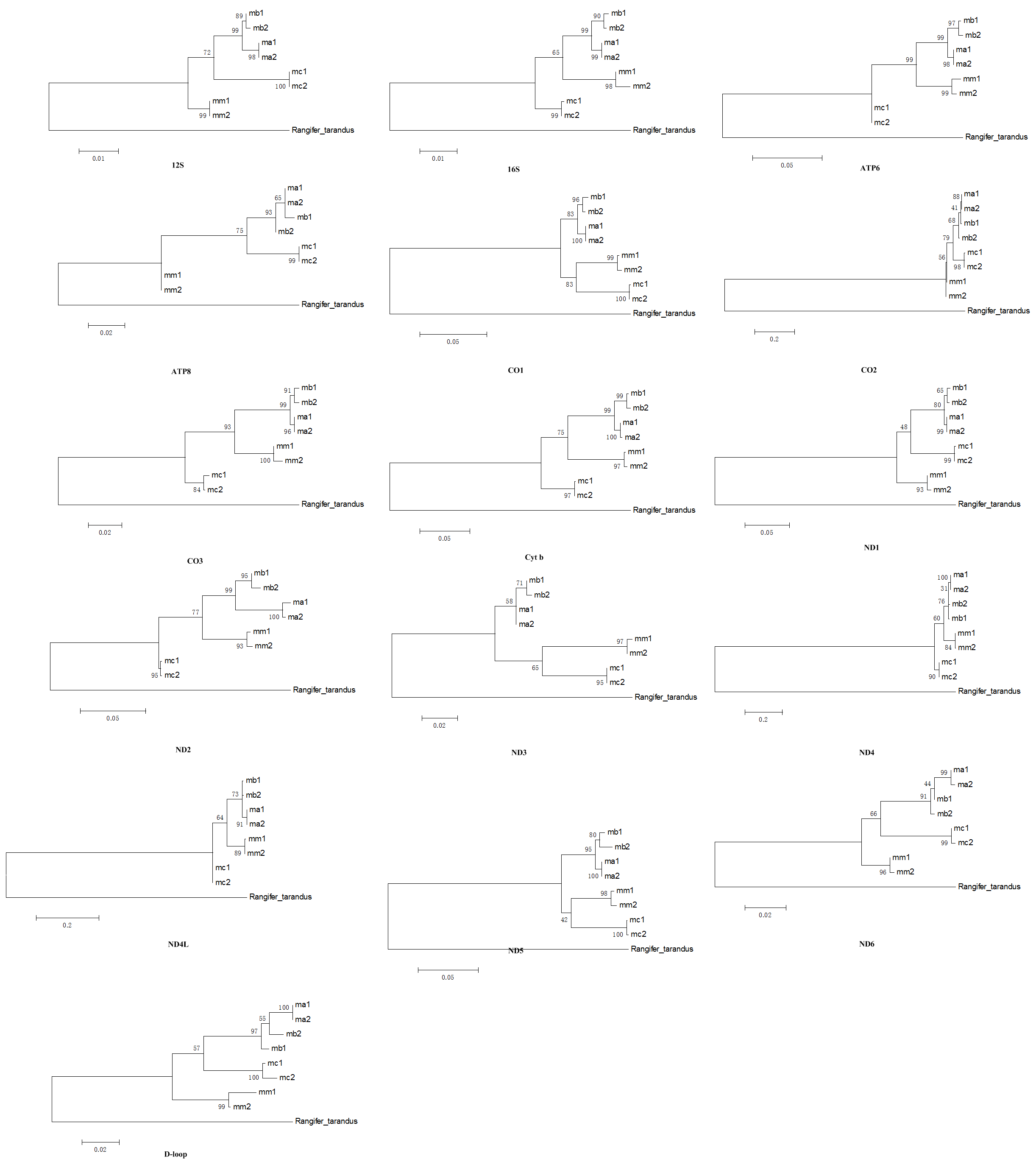

Supplement: S2 Fig — The values on nodes indicate Bootstrap support. (TIF) [file pone.0134183.s007.tif]

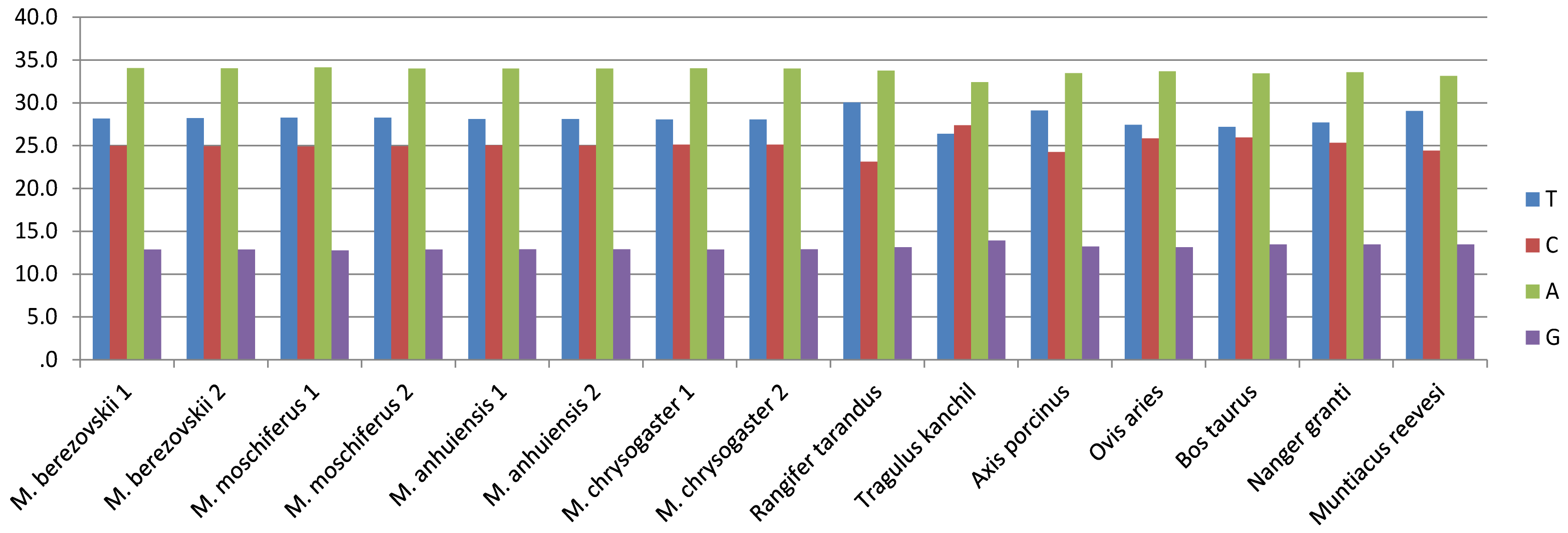

Supplement: S3 Fig — (TIF) [file pone.0134183.s008.tif]

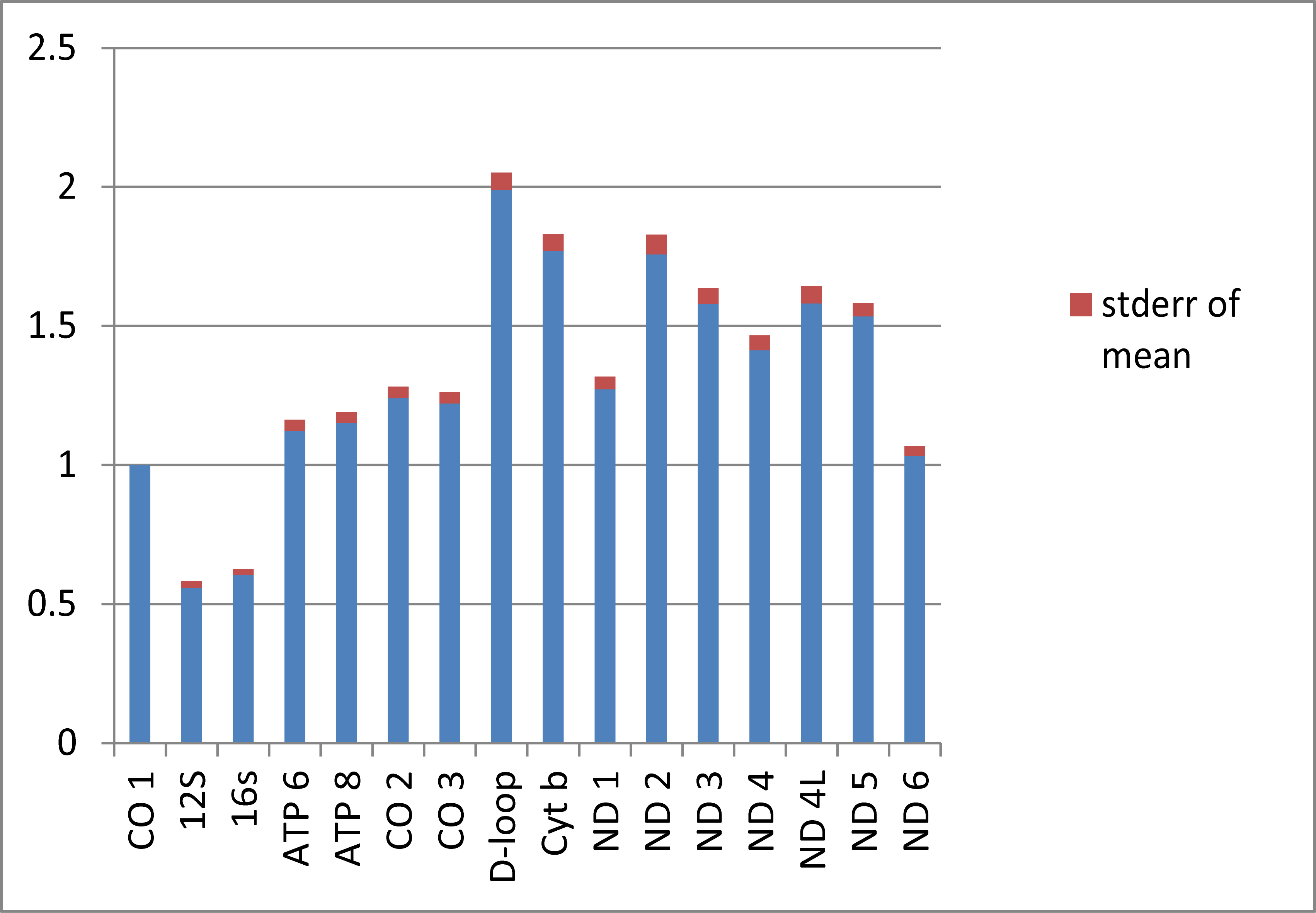

Supplement: S4 Fig — The relative evolution rate of CO1 was regard as the standard control value. (TIF) [file pone.0134183.s009.tif]
