# Supplementary material for: Species Delimitation in the Genus Moschus (Ruminantia: Moschidae) and Its High-Plateau Origin
Source: PLoS One. 2015 Aug 17;10(8):e0134183. doi: 10.1371/journal.pone.0134183 (PMC4539215; doi:10.1371/journal.pone.0134183)
Supplement: S1 Table — (DOC) [file pone.0134183.s010.doc]

Table S1. Taxa analyzed in the present study

| TAXA | Tissue origin | **Accession Number** | **Dataset** |
| --- | --- | --- | --- |
| SUB-ORDER RUMINANTIA |  |  |  |
| INFRA-ORDER PECORA |  |  |  |
| FAMILY BOVIDAE |  |  |  |
| Subfamily BOVINAE |  |  |  |
| Tribe BOVINI |  |  |  |
| *Bos taurus taurus* |  | EU177832 | Dataset 1, 4, 5 |
| **Tribe BOSELAPHINI** |  |  |  |
| *Boselaphus tragocamelus* |  | EF536350 | Dataset 1, 4, 5 |
| **Tribe TRAGELAPHINI** |  |  |  |
| *Tragelaphus imberbis* |  | EF536356 | Dataset 1, 4, 5 |
| **Subfamily ANTILOPINAE** |  |  |  |
| **Tribe ANTILOPINI** |  |  |  |
| *Nanger granti* |  | JN632666 | Dataset 1, 4, 5 |
| **Tribe CAPRINI** |  |  |  |
| *Ovis aries* |  | NC 001941 | Dataset 1, 4, 5 |
| *Pseudois nayaur* |  | FJ207537 | Dataset 1, 4, 5 |
| **FAMILY CERVIDAE** |  |  |  |
| **Subfamily CERVINAE** |  |  |  |
| **Tribe CERVINI** |  |  |  |
| *Axis porcinus* |  | JN632600 | Dataset 1, 4, 5 |
| **Tribe MUNTIACINI** |  |  |  |
| *Muntiacus reevesi* |  | NC 008491 | Dataset 1, 4, 5 |
| **Subfamily CAPREOLINAE** |  |  |  |
| **Tribe Odocoileini** |  |  |  |
| *Rangifer tarandus* |  | NC 007703 | Dataset 1, 4, 5 |
| **FAMILY MOSCHIDAE** |  |  |  |
| *Moschus anhuiensis* 1– Anhui musk deer | Huoshan, Anhui Province | NC020017 | Dataset 1, 2, 3, 4, 5 |
| *M. anhuiensis* 2– Anhui musk deer | Yuexi, Anhui Province | KP684124 | Dataset 1, 2, 3, 4, 5 |
| *M. berezovskii* 1*–* Dwarf musk deer |  | NC012694 | Dataset 1, 2, 3, 4, 5 |
| *M. berezovskii* 2*–* Dwarf musk deer |  | JQ409122 | Dataset 1, 2, 3, 4, 5 |
| *M. moschiferus* 1– Siberian musk deer |  | JN632662 | Dataset 1, 2, 3, 4, 5 |
| *M. moschiferus* 2– Siberian musk deer |  | NC013753 | Dataset 1, 2, 3, 4, 5 |
| *M. chrysogaster* 1– Alpine musk deer | Xinglong Mountain, Gansu Province | KC425457 | Dataset 1, 2, 3, 4, 5 |
| *M. chrysogaster* 2– Alpine musk deer | Qinghai Lake, Qinghai Province | KP684123 | Dataset 1, 2, 3, 4, 5 |
| *M. fuscus* – Tawny Musk Deer (12S rRNA) | unpublished | AY184427 | Dataset 1, 3 |
| *M. fuscus* – Tawny Musk Deer (Cyt *b*) |  | AF026888 | Dataset 1, 3 |
| *M. leucogaster* – Himalayan Musk Deer (12S rRNA) | unpublished | AY921577 | Dataset 1, 3 |
| *M. leucogaster* – Himalayan Musk Deer (Cyt *b*) |  | AF026889 | Dataset 1, 3 |
| **INFRA-ORDER TRAGULINA** |  |  |  |
| **FAMILY TRAGULIDAE** |  |  |  |
| *Tragulus kanchil* |  | JN632709 | Dataset 1, 4, 5 |

**References**

1. Achilli A, Olivieri A, Pellecchia M, Uboldi C, Colli L, Al-Zahery N, et al. (2008) Mitochondrial genomes of extinct aurochs survive in domestic cattle. Curr Biol 18: 157-158.

2. Hassanin A, Delsuc F, Ropiquet A, Hammer C, Jansen van Vuuren B, Matthee C, et al. (2012) Pattern and timing of diversification of Cetartiodactyla (Mammalia, Laurasiatheria), as revealed by a comprehensive analysis of mitochondrial genomes. CR Biol 335: 32–50.

3. Hiendleder S, Lewalski H, Wassmuth R, Janke A (1998) The complete mitochondrial DNA sequence of the domestic sheep (*Ovis aries*) and comparison with the other major ovine haplotype. J Mol Evol 47: 441-448.

4. Hassanin A, Ropiquet A, Couloux A, Cruaud C (2009) Evolution of the mitochondrial genome in mammals living at high altitude: new insights from a study of the tribe Caprini (Bovidae, Antilopinae). J Mol Evol 68: 293-310.

5. Peng HY, Liu SC, Zeng B, Zou FD, Zhang XY, Yue BS (2009) The complete mitochondrial genome and phylogenetic analysis of forest musk deer (Moschus berezovskii). J Nat Hist 43: 1219–1227.

6. Yang CZ, Xiao Z, Zou Y, Zhang XY, Yang B, Hao YH, et al. (2015) DNA barcoding revises a misidentification on musk deer. Mitochondrial DNA 26: 605-612.

7. Jang KH, Hwang UW (2010) Mitochondrial genome of the Korean musk deer *Moschus moschiferus* (Artiodactyla, Ruminantia, Moschidae). Mitochondrial DNA 21: 65-67.

8. Su B, Wang YX, Lan H, Wang W, Zhang YP (1999) Phylogenetic Study of Complete Cytochrome  *b* Genes in Musk Deer (Genus Moschus ) Using Museum Samples. Mol Phylogenet Evol 12: 241–249.
