# Supplementary material for: Species Delimitation in the Genus Moschus (Ruminantia: Moschidae) and Its High-Plateau Origin
Source: PLoS One. 2015 Aug 17;10(8):e0134183. doi: 10.1371/journal.pone.0134183 (PMC4539215; doi:10.1371/journal.pone.0134183)
Supplement: S2 Table — (DOC) [file pone.0134183.s011.doc]

S2 Table. **Overlap PCR primer pairs of the mitochondrial DNA in *Moschus* (DOC).**

| No. of  primer pair | Primer name | Primer sequences (5’-3’) | Product | Product size  (bp) |
| --- | --- | --- | --- | --- |
| 1 | 12S-L604  16S-H1462 | F: CTCAAAGGACTTGGCGGTGC  R: CATTTTCTGGACAACCAGCTATC | 12S rRNA*, Val, 16S rRNA* | 888 |
| 2 | 16S-L1372  16S-H2187 | F: CCGAAACCAGACGAGCTACT  R: CTCCATAGGGTCTTCTCGTC | 16S rRNA* | 833 |
| 3 | 16S-L2069  ND1-H2760 | F: CTGACCGTGCAAAGGTAGCA  R: GTTAAGGAGAGGATTTGAATCTC | 16S rRNA*, NADH1* | 728 |
| 4 | Leu-L2728  Met-H3887 | F: GTTAAGGTGGCAGAGCCCGG  R: CCGATAGCTTAATTAGCTGACC | Leu*, NADH1, Ile, Gln, Met* | 1201 |
| 5 | Ile-L3777  ND2-H4512 | F: GACAAAAGAGTTACTTTGATAGAG  R: AGGACTGCTGTTATTCAGCC | Ile*, Gln, Met, NADH2* | 779 |
| 6 | ND2-L4308  Ala-H5087 | F: TTCTGAGTCCCAGAAGTAACAC  R: GTGGTTGATTTGCATTCAATTGATG | NADH2*, Trp, Ala* | 828 |
| 7 | Trp-L5030  COI-H5741 | F: AGACCAAGAGCCTTCAAAGC  R: CACGGTTCAGCCTGTTCCTGC | Trp*, Ala, Asn, Cys, Tyr, COI* | 745 |
| 8 | COI-L5559  Ser-H6922  (COI-L6093**) | F: CAACGTAATTGTAACCGCACA  R: GGTTCGATTCCTTCCTTTCT  (F: CAACACTTATTCTGATTCTTTGG) | COI*, Ser* | 1403 |
| 9 | COI-L6531  ATP8-H7828 | F: CACTACGTATTATCAATAGGAGC  R: GTCACGTTGATGTGTCTAGTTG | COI*, Ser, Asp, COII, Lys, ATP8* | 1340 |
| 10 | COII-L7387  COIII-H8657 | F: GGACATCAATGATACTGAAGCTA  R: GGGTTTGGTGTGTCATTATGTG | COII*, Lys, ATP8, ATP6, COIII* | 1313 |
| 11 | ATP6-L8529  Gly-H9472 | F: GAGCCACACTTGCACTAATAAG  R: GAAACTAACTGATTGGAAGTCAG | ATP6*, COIII, Gly* | 986 |
| 12 | COIII-L9389  ND4-H10591 | F: AGCCGCTGCCTGATACTGAC  R: AGTGTGGGGACTAGTGTTGC | COIII*, Gly, NADH3, Arg,  NADH 4L, NADH4* | 1241 |
| 13 | ND4-L10466  Ser-H11660 | F: CTAATACTAATAGCTAGCCAACA  R: CTTGCATACTTTTTCGGTAAATAAG | NADH4*, His, Ser* | 1241 |
| 14 | ND4-L11270  ND5-H12297 | F: TAGCCCGAGGCCTACAAAC  R: ATTGCTTGTAAGGCTGCTGT | NADH4*, His, Ser, Leu, NADH5* | 1064 |
| 15 | ND5-L12080  ND5-H13097 | F: CAATAATATTTGTCCCAGTAGCA  R: GGAAATCGGGGTTGTCCTA | NADH5* | 1057 |
| 16 | ND5-L12950  Glu-H14176 | F: CAGCCTTGCACTAACAGGAAT  R: GTAGTTGAATGACTTCGATGGT | NADH5*, NADH6, Glu* | 1267 |
| 17 | ND6-L13696  H15149 † | F: AACTAATCAAGTACCATAACTGTA  R: AAACTGCAGCCCCTCAGAATGATATTTGTCCTCA | Glu, Cyt B* | 971 |
| 18 | L14724 †  H15915 † | F: CGAAGCTTGATATGAAAAACCATCGTTG  R: AACTGCAGTCATCTCCGGTTTACAAGA | Glu*, Cyt B, Thr* | 1247 |
| 19 | Cytb -L15270  CR-H16118 | F: ATCATTATTGGACAACTAGCATC  R: TGGTTTCACGAGGCATGGT | Thr, Pro, CR* | ~635 |
| 20 | Pro-L15461f  Phe-H60r | F: CACCGTCAACACCCAAAGCTG  R: AGCTAGTACACTCATCTAGGC | Pro *, CR, Phe* | ~1026 |
| 21 | CR-L16442  12S-H748 | F: GGACTTAACTGCATCTTGAGC  R: AGGTTACACCTTGACCTAACG | CR*, Phe, 12S rRNA* | ~1205 |
