# Supplementary material for: Species Delimitation in the Genus Moschus (Ruminantia: Moschidae) and Its High-Plateau Origin
Source: PLoS One. 2015 Aug 17;10(8):e0134183. doi: 10.1371/journal.pone.0134183 (PMC4539215; doi:10.1371/journal.pone.0134183)
Supplement: S3 Table — (DOCX) [file pone.0134183.s012.docx]

**Table S2** Calibration points of the main splitting events within Cetartiodactyla [[1](#_ENREF_1)].

|  | BD-SOFT Mean | SD | BD-HARD Mean | SD | UNI-HARD Mean | SD | Geologic Period |
| --- | --- | --- | --- | --- | --- | --- | --- |
| Tragulidae | 21.8 | 6.6 | 22.5 | 7.2 | 24.3 | 6.2 | Late Oligocene–Early Miocene |
| Antilopinae | 17.0 | 1.8 | 17.5 | 2.2 | 19.6 | 0.8 | Early Miocene–Middle Miocene |
| Bovinae | 16.4 | 2.0 | 16.8 | 2.2 | 18.9 | 1.1 | Early Miocene–Middle Miocene |
| Giraffidae | 15.2 | 2.9 | 15.7 | 3.3 | 17.2 | 3.3 | Early Miocene–Middle Miocene |
| Bovini | 13.3 | 1.9 | 13.8 | 2.4 | 16.1 | 1.6 | Middle Miocene |
| Antilopini | 12.02 | 1.6 | 12.5 | 2.2 | 15.1 | 1.3 | Middle Miocene |
| Odocoileinae | 9.2 | 0.8 | 9.3 | 0.9 | 10.0 | 1.1 | Late Miocene |
| Caprini | 9.2 | 1.8 | 9.8 | 2.3 | 12.5 | 1.4 | Late Miocene |
| Reduncini | 9.1 | 1.4 | 9.6 | 1.9 | 12.0 | 1.8 | Late Miocene |
| Cephalophini | 8.9 | 1.2 | 9.8 | 2.1 | 12.3 | 1.6 | Late Miocene |
| Cervinae | 8.8 | 0.9 | 9.0 | 0.9 | 9.1 | 0.9 | Late Miocene |
| Neotragini | 8.8 | 1.5 | 9.4 | 2.1 | 11.1 | 2.0 | Late Miocene |
| Muntiacini | 7.4 | 0.9 | 7.6 | 1.0 | 7.7 | 1.0 | Late Miocene |
| Tragelaphini | 6.8 | 1.7 | 7.2 | 2.0 | 9.1 | 2.1 | Late Miocene |
| Hippotragini | 6.8 | 1.6 | 7.3 | 2.2 | 9.8 | 1.8 | Late Miocene–Pliocene |
| Boselpahini | 6.6 | 1.8 | 6.9 | 2.1 | 8.8 | 2.4 | Late Miocene–Pliocene |
| Alcelaphini | 6.0 | 1.5 | 6.6 | 2.0 | 8.8 | 1.9 | Late Miocene–Pliocene |
| Cervini | 5.9 | 0.8 | 6.1 | 1.0 | 6.3 | 1.0 | Miocene/Pliocene limit |
| Capreolini | 5.6 | 0.7 | 5.6 | 0.8 | 6.0 | 1.0 | Miocene/Pliocene limit |

Note: BD: Birth-Death; UNI: uniform; SD: standard deviation.

1. Hassanin A, Delsuc F, Ropiquet A, Hammer C, Jansen van Vuuren B, et al. (2012) Pattern and timing of diversification of Cetartiodactyla (Mammalia, Laurasiatheria), as revealed by a comprehensive analysis of mitochondrial genomes. Comptes rendus biologies 335: 32-50.
