# Supplementary material for: Species Delimitation in the Genus Moschus (Ruminantia: Moschidae) and Its High-Plateau Origin
Source: PLoS One. 2015 Aug 17;10(8):e0134183. doi: 10.1371/journal.pone.0134183 (PMC4539215; doi:10.1371/journal.pone.0134183)
Supplement: S4 Table — Model of evolution was calculated based on the gene in Mouchus. (DOCX) [file pone.0134183.s013.docx]

**Table S3 Comparison of the model of evolution used in Bayesian analyses, overall base frequencies, AT skew, and GC skew.** Model of evolution was calculated based on the gene in *Mouchus*.

| Locus | Model of evolution | Base frequencies | | | | AT skew | GC skew |
| --- | --- | --- | --- | --- | --- | --- | --- |
|  |  | A | C | G | T |  |  |
| CO1 | GTR+G | 29.2 | 23.0 | 16.0 | 32.0 | - | - |
| 12S | GTR+I | 36.9 | 23.0 | 17.2 | 22.9 | - | - |
| 16S | GTR+I | 38.1 | 20.1 | 16.9 | 24.9 | - | - |
| ATP6 | HKY+G | 32.4 | 27.0 | 10.7 | 30.0 | - | - |
| ATP8 | HKY+I | 41.2 | 23.0 | 6.20 | 29.6 | - | - |
| CO2 | HKY+G | 36.4 | 23.5 | 12.0 | 28.2 | - | - |
| CO3 | GTR+I | 26.9 | 28.1 | 14.5 | 30.6 | - | - |
| D-loop | HKY+I | 32.0 | 22.00 | 13.6 | 32.4 | - | - |
| Cyt b | GTR+G | 32.1 | 26.0 | 12.6 | 29.4 | - | - |
| ND1 | GTR+I+G | 32.7 | 25.7 | 11.4 | 30.2 | - | - |
| ND2 | GTR+G | 37.5 | 26.0 | 8.0 | 28.5 | - | - |
| ND3 | HKY+I | 31.6 | 28.4 | 10.9 | 29.1 | - | - |
| ND4 | GTR+I+G | 33.1 | 27.2 | 9.8 | 29.8 | - | - |
| ND4L | HKY+G | 32.0 | 24.9 | 11.0 | 32.2 | - | - |
| ND5 | GTR+G | 34.0 | 26.3 | 10.2 | 29.5 | - | - |
| ND6 | GTR+G | 42.6 | 27.3 | 6.8 | 23.4 | - | - |
| M．berezovskii 1 | - | 34.1 | 25.0 | 12.8 | 28.1 | 0.0965 | 37.8 |
| M．berezovskii 2 | - | 34.0 | 24.9 | 12.9 | 28.2 | 0.0932 | 37.8 |
| M. moschiferus 1 | - | 34.1 | 24.9 | 12.8 | 28.2 | 0.0947 | 37.7 |
| M. moschiferus 2 | - | 34.0 | 24.9 | 12.8 | 28.3 | 0.0915 | 37.7 |
| M. anhuiensis 1 | - | 34.0 | 25.0 | 12.9 | 28.1 | 0.0950 | 37.9 |
| M. anhuiensis 2 | - | 34.0 | 25.0 | 12.9 | 28.1 | 0.0950 | 37.9 |
| M. chrysogaster 1 | - | 34.0 | 25.1 | 12.8 | 28.0 | 0.0968 | 37.9 |
| M. chrysogaster 2 | - | 34.0 | 25.1 | 12.9 | 28.0 | 0.0968 | 38.0 |
| Rangifer tarandus | - | 33.7 | 23.1 | 13.1 | 30.0 | 0.0581 | 36.2 |
| Tragulus kanchil | - | 32.4 | 27.4 | 13.9 | 26.4 | 0.1020 | 41.3 |
| Axis porcinus | - | 33.5 | 24.2 | 13.2 | 29.1 | 0.0703 | 37.4 |
| Ovis aries | - | 33.7 | 25.8 | 13.1 | 27.4 | 0.1031 | 38.9 |
| Bos taurus | - | 33.4 | 26.0 | 13.5 | 27.2 | 0.1023 | 39.5 |
| Nanger granti | - | 33.5 | 25.3 | 13.5 | 27.7 | 0.0948 | 38.8 |
| Muntiacus reevesi | - | 33.1 | 24.4 | 13.5 | 29.0 | 0.0660 | 37.9 |
